# Singing and social inclusion

**DOI:** 10.3389/fpsyg.2014.00803

**Published:** 2014-07-29

**Authors:** Graham F. Welch, Evangelos Himonides, Jo Saunders, Ioulia Papageorgi, Marc Sarazin

**Affiliations:** ^1^Department of Culture, Communication and Media, International Music Education Research Centre, Institute of Education, University of LondonLondon, UK; ^2^Department of Social Sciences, University of NicosiaNicosia, Cyprus; ^3^Department of Education, University of OxfordOxford, UK

**Keywords:** singing, development, *Sing Up*, self-concept, social inclusion, children

## Abstract

There is a growing body of neurological, cognitive, and social psychological research to suggest the possibility of positive transfer effects from structured musical engagement. In particular, there is evidence to suggest that engagement in musical activities may impact on social inclusion (sense of self and of being socially integrated). Tackling social exclusion and promoting social inclusion are common concerns internationally, such as in the UK and the EC, and there are many diverse Government ministries and agencies globally that see the arts in general and music in particular as a key means by which social needs can be addressed. As part of a wider evaluation of a national, Government-sponsored music education initiative for Primary-aged children in England (“Sing Up”), opportunity was taken by the authors, at the request of the funders, to assess any possible relationship between (a) children's developing singing behavior and development and (b) their social inclusion (sense of self and of being socially integrated). Subsequently, it was possible to match data from *n* = 6087 participants, drawn from the final 3 years of data collection (2008–2011), in terms of each child's individually assessed singing ability (based on their singing behavior of two well-known songs to create a “normalized singing score”) and their written responses to a specially-designed questionnaire that included a set of statements related to children's sense of being socially included to which the children indicated their level of agreement on a seven-point Likert scale. Data analyses suggested that the higher the normalized singing development rating, the more positive the child's self-concept and sense of being socially included, irrespective of singer age, sex and ethnicity.

## Introduction

According to their published policies, one of the major concerns of many contemporary Governments and international organizations is social cohesion. The United Nations' policy on “sustainable development,” for example, has social inclusion as a prime objective for the “wellbeing of individuals and societies” (UN General Assembly, 2013, p. 12). In Australia, a *Social Inclusion Board* was appointed during the period 2008–2013 to generate regular reports on the Government's stated commitment to supporting increased social inclusion (Australian Social Inclusion Board, 2010). In the European Union (EU), the *Social Protection Committee of the European Commission* continues to be an advisory policy committee that monitors social conditions in the EU and produces annual reports. Recently, this EC Committee commented that “the social situation in the EU is worsening” in 2012, with nearly one quarter of the population “at risk of poverty or social exclusion in the EU” (2013, p. 8). This equates to 120 million people (Agilis, 2012) and includes 27% of all children in Europe and 20.5% of those over 65.

In a related policy initiative, EC Member States established an *EU Network of Independent Experts on Social Inclusion* to evaluate each country's social aspects of their official National Reform Programmes (NRPs). The EU Network's initial report (Frazer and Marlier, 2011, pp. 5–7) suggested that “in many instances, the social inclusion measures proposed in the NRPs are imprecise or aspirational in nature… Many Member States still need to develop better targeted social inclusion measures and to develop specific strategies to reach the most vulnerable.”

Social *exclusion* is defined as “… a process whereby certain individuals are pushed to the edge of society and prevented from participating fully by virtue of their poverty, or lack of basic competencies and lifelong learning opportunities, or as a result of discrimination” (EC, 2009). In contrast, social *inclusion* is “… a process which ensures that those at risk of poverty and social exclusion gain the opportunities and resources necessary to participate fully in economic, social, and cultural life and to enjoy a standard of living and wellbeing that is considered normal in the society in which they live” (EC, *op.cit.*). In the UK, for example, 26% of the total child population in 2006 were considered to be at risk of poverty (Eurostat—EU-SILC, 2006), based on data provided by the UK Government and with the implication that such risk would impact negatively on health, education and life expectancy. By 2012, 18% of UK children were reported to be living in households with relatively low income, associated with limited access to household materials, joblessness and social deprivation (EC, 2013), and with 120,000 families experiencing multiple problems (DWP, 2012).

In terms of schooling, social inclusion is seen as important in an educational context because schools are expected to facilitate the process whereby all children are able to access and be successful in terms of the education programme being offered and also to use this as a basis for their current and future full engagement with the wider social culture (Rosenberg, 1989; Frederickson and Furnham, 2001; Alexiadou, 2002; Mannion, 2003). From a sociological perspective, therefore, poverty should be considered to be an important potential contributor to an individual's sense of social exclusion. Also implicated are the social structures and values inherent in society's systems of relative social power (Allman, 2013), such as embedded in educational policies and organizational behaviors.

Despite such challenges, there is also a range of research evidence to suggest that social disadvantage can be overcome, especially where the home and school environments are nurturing. For example, academic progress up to the first years of secondary school that defies the odds of disadvantage is stimulated in homes where parenting is a process of “active cultivation” (Siraj-Blatchford et al., 2011, p. 70). This facilitates and nurtures children's cognitive and social skills, thus allowing children to benefit from what the educational system has to offer, such as provided in good or excellent quality pre-school settings, a combination that promotes resilience in the face of adversity (cf. O'Dougherty Wright et al., 2013).

Given the political emphasis on fostering social inclusion, it is not surprising that there have been a number of related initiatives to address this concern, including one strand that has focused on intervention through engagement in the arts. In the U.S., for example, the National Endowment for the Arts in collaboration with the U.S. Department of Health & Human Services recently commissioned a report into “the arts and human development” (Hanna et al., 2011). This followed a “national showcasing” in Washington of U.S. programmes that were reported to provide evidence of positive cognitive, social, and behavioral outcomes from arts interventions for different age groups, such as children in low-income and “at risk” families (Brown et al., 2010), adolescents (Catterall, 2009) and older adults (Noice and Noice, 2009).

A similar research-based public advocacy-related document emerged from evaluative research in New South Wales, Australia (Vaughan et al., 2011). The investigation included both a systematic review of previous research studies, as well as the collection and synthesis of new evaluative data on underserved communities. The summative findings illustrated the association of arts and music programmes on a wide range of behaviors, including academic achievement (English, mathematics, science and technology) and social-emotional wellbeing. In England, the Government's “National Plan for Music Education” (DFE, 2011) cites evidence of the social value of music as part of its rationale for promoting the national provision of systematic and high quality music education in the lives of all children and young people. Elsewhere, other studies have reported positive impacts of musical activities on social development, such as an enhanced self-concept for pupils in lower socio-economic settings (cf. Catterall et al., 1999), and increased positive social-emotional capacities, such as empathy (Rabinowitch et al., 2013).

Music's social benefits are also reported as an outcome of various adult music programmes, such as in the study of choral activity with a group of homeless men in Canada (Bailey and Davidson, 2002), the social bonding of male singers in Iceland (Faulkner and Davidson, 2006), and in a New Zealand-based cross-cultural study of the apparent universality of the social bonding function of music, such as through shared music preferences (Boer, 2009). There is also an extensive research-based literature emerging into the impact of music participation on wellbeing and health in the elderly (e.g., Teater and Baldwin, 2014; see Clift and Hancox, 2010; Creech et al., 2013b for reviews). Self-reported benefits are evidenced in a Korean study of undergraduate non-vocalists (Chong, 2010). Almost all (98%) of the participants enjoyed singing (although 8% preferred to do this when alone). The reasons cited for their enjoyment of singing included self-expression, esthetic experience, interpersonal relation- ships, stress reduction/mood change, spirituality, empowerment/identity, and self-actualization.

Additionally, there is also emerging evidence from the field of neuroscience concerning social behavior, such as related to mapping the underlying neural mechanisms that support social behaviors and attachment (e.g., Nelson et al., 2005; Bartz and Hollander, 2006; Cirelli et al., 2013). Related literature reviews propose a neurobiological role for music in fostering integration through communal music activity (Freeman, 2000) and in the psychology of well-being (Croom, 2012), believed to be related to what is currently known concerning the underlying neurochemistry of music (Chanda and Levitin, 2013). A concept of “empathic creativity” has been used to highlight the psychological power of successful music making with others where empathy and creativity are core attributes (Cross et al., 2012).

Concerning the younger generation, one global international organization whose stated mission emphasizes social inclusion through a raft of diverse music programmes is *Jeunesses Musicales International*, with member organizations in 45 countries (JMI, 2010). Another, slightly more recent and widespread global initiative with social inclusion at its core relates to the accrual of social benefits emerging from children's and young people's collective instrumental learning, both within and inspired by the *El Sistema* programme in Venezuela. “A core aim of *El Sistema* is to effect social change through the provision of musical and intellectual opportunities for young people from poor and vulnerable communities who would not otherwise access such experiences.” (Creech et al., 2013a, p. 17). This recent review estimated that there are at least 277 *Sistema* and *Sistema*-type programmes across 58 countries, with widespread reports that this particular approach to instrumental music learning supports social, emotional, and cognitive wellbeing through, *inter alia*, the acquisition of social capital and the development of interpersonal relationships, linked to a sense of collective and shared purpose—the orchestra as a community. One example is the Orchestra of the Americas for Social Inclusion (known as OASIS Caribbean), an orchestra programme for young people considered to be “at risk” in the Caribbean region (Harvey and McNeilly, 2012).

The use of the lens of social capital theory is also evidenced in its application in other studies of the social impact of arts participation. This theory considers, *inter alia*, intra- and inter-familial relationships, as well as their communal interactions, and the possible influences on health and wellbeing. A recent systematic review of twenty-two peer-reviewed studies concluded that, after poverty, social capital (e.g., within the family and community) was one of the best predictors of children's welfare, having an impact on mental and physical health, educational attainment and labor-market participation (Ferguson, 2006). However, another study argued that not all participants gain equally from arts participation in terms of social inclusion. This smaller scale, short-term longitudinal study examined of a group of children participating communally in a UK Government flagship singing programme (*Sing Up*) in NE England (Hampshire and Matthijsse, 2010). The authors report that most of the children benefited from the experience, with participation being instrumental in improving emotional and social wellbeing, and providing opportunities to develop social capital. For a minority, however, being members of a communal music programme outside their immediate peer and friendship groups posed risks to their established social networks.

Other research that reports findings of particular social benefits accruing through participation in music includes evidence that sustained, formal piano lessons can support the development of children's self-esteem (Costa-Giomi, 2004). Similar findings were reported in an intervention study with controls of specialized school-based music classes in Australia (Rickard et al., 2013) and Israel (Portowitz et al., 2009). Music is also reported to have beneficial impacts on health and psychosocial wellbeing in a wide range of other, non-school contexts and across the lifespan (cf. MacDonald et al., 2012; MacDonald, 2013). These include hospitals (Preti and Welch, 2011; Preti, 2013), prisons (Henley et al., 2012), young offender institutions (Smeijesters et al., 2011; Barrett and Baker, 2012) and choral settings (Langston and Barrett, 2008; Dingle et al., 2013).

One novel approach was evidenced in an EC-funded study across four countries (Finland, England, Switzerland and Greece) that investigated how to use the attractiveness and power of new mobile phone technology and children's widespread interest in music to create a new tool that would enable children to have fun making music, whilst improving their knowledge and skills, and fostering their sense of social inclusion (Fredrikson et al., 2009; Myllykoski and Paananen, 2009). The research was targeted at particular groups of children at risk of social exclusion, particularly those with moderate learning difficulties or who were newly immigrant to their host communities. As part of the evaluation phase of the project's social inclusion research tools, data emerged from participants (*n* = 110) drawn from four schools (two in England and two in Finland) to indicate that the higher the number of days per week that the children either played a musical instrument or sang with their friends, the more likely they were to report themselves as socially included (Rinta et al., 2011a).

One integral facet of an individual's sense of being socially included (or not) is that this perception is interwoven with their overall self-concept, such as how they view themselves (self-esteem, e.g., Rosenberg, 1989), their personal sense of agency in getting things done (self-efficacy, e.g., Bandura, 1997) and of their self-regulation and communication skills (e.g., Frederickson and Furnham, 2001). Links between self-concept and social inclusion are also theorized as integral to Ryan and Deci's (2000) self-motivation theory, based on three basic categories of psychological needs: competence, a sense of relatedness to others and an increasing sense of autonomy. Each of these aspects of self has a musical correlate as part of an individual's self-view. For example, self-efficacy is important to both persistence and achievement in music (Eccles et al., 1993; Schmidt et al., 2006; Ritchie and Williamon, 2011), whilst McPherson and McCormick (2006) also report direct links between self-efficacy and measures related to the study of music, such as time spent in formal and informal practice, self-regulation in practice, and overall music achievement level. Furthermore, social interaction during music making activities is reported to play an essential role in facilitating social and musical development (Young, 2003; Wiggins, 2007). Such interaction is a common feature of early childhood, where musical games and music-based rituals between caregivers and infants are a major source of building up supportive and healthy social attachments, as well as for stimulating language and intellectual development (e.g., Papousek, 1996; Dissanayake, 2008; Trevarthen, 2008).

As can be seen from the various studies reported above, there have been considerable national and international policy initiatives to foster social inclusion, as well as a wide range of related studies investigating the potential and actual social benefits of arts and music-based interventions. In an associated policy initiative, the (then) UK Government initiated a “Music Manifesto” in 2004, sponsored by the Ministries for Education (DCSF) and Culture, Media and Sport (DCMS) to campaign “to ensure that all children and young people have access to high quality music education^1^.” Under the umbrella of the “Music Manifesto” in England (Music Manifesto, 2006), one major initiative focused on Primary school-aged children's singing. A four-year, £40 m National Singing Programme *Sing Up* was officially launched in November 2007 with the intention of ensuring that singing became part of Early Years and Primary education for all children in England by 2012^2^, a cultural programme initiative that was designed to link, in part, to wider preparations for the London-based Olympic Games. Prior to the official launch, a team from the Institute of Education, University of London, led by the first author, was appointed to undertake a research evaluation of key elements of the *Sing Up* Programme. The agreed research focus was primarily on whether the various strands of the *Sing Up* programme in combination (which included, for example, workforce development for teachers and community musicians, the development of a web-based song bank resource, an awards programme for schools, as well as initiatives involving school-based collaboration with singing specialists) were impacting positively on (a) the singing behavior and development of the participant children and (b) the children's attitudes toward singing (an aspect of the research that is not reported here in this article because of its length requirements). In subsequent discussions, the funders also requested an additional focus. This concerned (c) a measure of children's self-concept and sense of being socially included in order to seek a possible indicator of a specific “wider benefit” that might arise from being involved in the National Singing Programme.

There is a growing body of evidence to suggest that children's singing behavior is subject to developmental processes that are mediated by maturation, experience and socio-cultural context (e.g., Welch, 1986, 2006, 2009; Welch et al., 1997). In general, older children tend to be more skilled in their singing behaviors, such that only a small minority continue have difficulty in singing in-tune by the age of eleven. Progress in singing competency is observable through significant changes in singing behavior and these changes can be mapped on the basis of published developmental frameworks of children's singing, grounded in empirical data (cf. Rutkowski, 1997; Welch, 1998; Mang, 2006). Such research-based studies recognize that a child that can become significantly more accomplished in their singing in a socially nurturing singing environment.

A review of the literature on social inclusion (Rinta et al., 2011b) reported that social inclusion is a multifaceted concept that embraces an interconnection between sociological and psychological factors (e.g., Atkinson et al., 2002; Micklewright, 2002; Baumeister et al., 2005; Twenge et al., 2007). Accordingly, the current study drew on this multifaceted conception to investigate aspects of social inclusion by drawing on related aspects of children's social self-concept. These embraced statements related to self-esteem (drawing on the work of Fitts, 1964; Rosenberg, 1989; Thornberry et al., 1994), self-efficacy (Nowicki and Strickland, 1973; Nowicki and Walker, 1973; Vispoel, 1994) and children's sense of being socially integrated (Fitts, 1964; Haeberlin et al., 1989; Achenbach, 1991), i.e., a broad pedagogical definition of pupils' sense of being socially included that embraced a personal self-view in the context of others. For the purposes of the current article, the term social inclusion is being treated as one concept that draws on sense of self and of being socially integrated.

This additional research focus was woven into the design of the participant children's attitudinal data collection to explore its possible relationship with their assessed singing behavior. Overall, the key research question that is the focus for this paper was: Is there any wider attitudinal benefit in terms of the children's self-concept and sense of being socially included being evidenced in relation to data on the same children's individually assessed song singing behavior and development?

## Materials and methods

Across the four years of *Sing Up* (2007–2011), the Institute of Education research team visited 184 schools nationally and collected individual singing data from 11,258 children. Some of the children were seen more than once as part of a longitudinal focus, resulting in a total of 13,096 assessments of individual children's singing being made. Schools were selected on the basis of being located in major cities and adjacent populations areas, supplemented by schools in other urban, suburban and rural settings. Choice was guided through professional contacts with Local Authority music advisors and university music education colleagues who were asked for advice on possible participant schools, the intention being to draw on local knowledge to ensure that a diverse range of school singing “cultures” were accessed, that is, schools with a known history of good singing and those without. There were also a number of Cathedral Choir Schools that were contacted directly.

In terms of ethical procedures, all participants (headteachers, teachers and pupils) had the purpose of the assessment explained in advance and this was in writing to the school using a specially prepared leaflet that was designed to use language in an age-appropriate way for the children. Each child was provided with a copy of the leaflet prior to participation for themselves and their parents and the school also took responsibility for explaining the research purpose and process, in line with English law where headteachers are empowered to act on behalf of the children's parents, a responsibility that is normally confirmed in writing by the parents/carers at the beginning of each school year. Under our ethical guidelines (based on BERA, 2004) we guaranteed anonymity to all participants and told them that they were allowed to withdraw from the assessment process at any time that they felt uncomfortable, for any or no reason. Participation was invited and not compulsory and children could ask to opt out if they did not wish to have their singing assessed.

Children's singing behavior and development were assessed individually by the application of a specially designed protocol. As well as aspects of children's spoken pitch and vocal range, the protocol required a member of the research team to assess each individual child's performance of two well-known songs against two established rating scales of singing development (Rutkowski, 1997; Welch, 1998—see Mang, 2006; Welch et al., 2012a for more detail) and combining the resultant data into a “normalized singing score” out of 100 to facilitate easy comparison between children. In essence, a normalized singing score of 95+ indicated that a child was able to sing the two focus songs in-tune, with accurate rhythm, pitch and lyrics across an extended singing range. Singing development was assessed by noting any changes in singing ability (as measured) principally by comparison with children of the same and different ages.

Data were collected from approximately equal numbers of girls (52%) and boys (48%), with 1:4 children coming from ethnic minority groups (i.e., in line with the proportions in official DCSF/DCMS statistics data for the population demographics of English Primary schools). Overall, as an intended part of the research design, 95% of the assessed children were aged from the age range 7+ to 10+ years. If the class teacher felt that support was needed with reading and understanding in the questionnaire task (see below), help was provided by the teacher and/or the child's usual teaching assistants.

The research process embraced two main sub-categories of participants within the overall data set, i.e., (i) children with experience of *Sing Up* initiatives at the time of their singing assessment (equating to 69% of the final dataset)^3^ and (ii) children without any *Sing Up* experience at the time of their assessment (31% of the final dataset)^4^.

Children were visited at their schools and their singing behaviors were assessed individually in a quiet, familiar space identified by the school's headteacher and/or music coordinator. Each child was taken through the assessment protocol, normally being tested individually within a small group of between 2 and 4 children that was drawn from the class. This allowed the other members of the group to observe and see what was required, as this had been shown previously to be an appropriate method of accessing better quality responses than individual testing alone (cf. Plumridge, 1972). Children tended to be less nervous and, if shy, able to understand more clearly what was expected of them by listening to their peers in advance. To avoid the effects of vocal modeling, no starting pitch was given for the song items and, although the member of the research team provided verbal encouragement to the child, they did not offer any sung prompt (cf. as advised by Mang, 2006). All children completed the assessments and none were excluded from the study.

The large numbers of participants necessitated a team-based approach to the data collection. Consequently, at the beginning of the research process, initial fieldwork was designed to allow moderation of team members' judgments. Members of the research team underwent initial training on sampled items in the assessment protocol, then completed a school visit in pairs prior to making visits on their own. The validity and ease of use of the assessment protocol was established through a short piloting process prior to commencement of the main data collection. The piloting process involved two members of the research team visiting a local Primary school and using the draft protocol to make digital audio recordings of individual children of different ages. The resultant vocal products were then put online, duplicated and randomized and then rated by both themselves and other members of the team according to the two assessment scales for singing behavior and development (Rutkowski, 1997; Welch, 1998). The rating results were compared statistically and this revealed a close agreement amongst the team members [Kendall's Coefficient of Concordance, *W*_(5, 19)_ = 0.91, *p* < 0.0001].

In addition to the individual singing assessment, all participant children were also asked to complete a specially designed questionnaire that explored their attitudes to various aspects of singing^5^ (Table 1). From the second year of data collection (2008–2009), the questionnaire was revised at the funder's request to include an additional measure of possible other-than-musical (= “wider”) benefit. The research team sought to measure any possible relationship in participants' social self-concept (sense of self and of being socially included) and their singing ability, noting that published commentaries on the “benefits” of singing include reports on positive social outcomes of choral activities on the individual (cf. Chorus America, 2003; Faulkner and Davidson, 2006; Clift et al., 2008). Accordingly, interwoven with 45 statements concerning children's attitudes to singing were 15 statements that related to aspects of children's social inclusion (sense of self and of being socially integrated). The 15 statements were based on a variety of sources to investigate different facets of children's social self-concept. These embraced statements related to self-esteem (Fitts, 1964; Rosenberg, 1989; Thornberry et al., 1994), self-efficacy (Nowicki and Strickland, 1973; Nowicki and Walker, 1973; Vispoel, 1994), as well as statements concerning children's sense of being socially integrated (Fitts, 1964; Haeberlin et al., 1989; Achenbach, 1991). Collectively, these 15 statements are interpreted as being related to children's social concept, hence the labeling here as sense of self and of being socially included.

**Table 1:** **National Singing Programme *Sing Up* Questionnaire with themes, 60 Questions**.

**S No.**	**Question text**	**Theme**
1	I sing at school	Singing at school
2	Singing at school will make me a better singer	Singing at school
3	I think that we should sing more at school	Singing at school
4	I feel good about myself	Social inclusion
5	I have sung in a performance at school	Singing at school
6	The boys in my class are better singers than the girls	Singing at school
7	I like the songs that I sing at school	Singing at school
8	The songs we sing at school are boring	Singing at school
9	The songs that I sing outside school are very different to the songs that I sing in school	Singing at school
10	I have control over my future	Social inclusion
11	I would like to sing a solo at school	Singing at school
12	My teacher taught me to sing	Singing at school
13	I like making music	Identity as a singer (emotional connection with singing)
14	Singing is fun	Identity as a singer (emotional connection with singing)
15	I think that hard work is more important than good luck	Social inclusion
16	Making music is fun	Identity as a singer (emotional connection with singing)
17	I like listening to music	Identity as a singer (emotional connection with singing)
18	School music is boring	Singing at school
19	I can learn to be a better singer at home	Singing at home
20	I feel that I am equal to everyone else	Social inclusion
21	I have many friends	Social inclusion
22	I learn songs at home	Singing at home
23	Members of my family tell me I am a good singer	Singing at home
24	I sing songs when I am in my room	Singing at home
25	I sing with my family	Singing at home
26	I am unable to do things as well as most other people	Social inclusion
27	I sing songs at home	Singing at home
28	My mother taught me to sing	Singing at home
29	My friends teach me songs	Singing in informal settings
30	I like singing with my friends	Singing in informal settings
31	Every time I try to get ahead something or somebody stops me	Social inclusion
32	I like singing in the playground	Singing in informal settings
33	Most of the songs I know I have learnt from the radio	Singing in informal settings
34	Most of the songs I know I have learnt from a CD	Singing in informal settings
35	I find singing easy	Identity as a singer (self)
36	My plans hardly ever work out	Social inclusion
37	I have a good singing voice	Identity as a singer (self)
38	I am the best singer in the class	Identity as a singer (self)
39	I can't sing	Identity as a singer (self)
40	Someone has told me that I can't sing	Identity as a singer (self)
41	I feel connected to my classmates	Social inclusion
42	On the whole, I am satisfied with myself	Social inclusion
43	I know I sing “out of tune”	Identity as a singer (self)
44	I know how my voice works	Identity as a singer (self)
45	I find it easier to learn a song when I see the notes written down	Identity as a singer (self)
46	I feel confident singing one voice of a two-voice song (in harmony)	Identity as a singer (self)
47	I feel useless at times	Social inclusion
48	Singing is a talent	Identity as a singer (self)
49	Singing is something that everyone can do	Identity as a singer (self)
50	I sing to express how I feel	Identity as a singer (emotional connection with singing)
51	I sing when I am happy	Identity as a singer (emotional connection with singing)
52	Sometimes I think I am no good at all	Social inclusion
53	I sing when I am sad	Identity as a singer (emotional connection with singing)
54	Singing makes me feel happy	Identity as a singer (emotional connection with singing)
55	Singing is something that I really enjoy doing	Identity as a singer (emotional connection with singing)
56	I prefer to sing when I am on my own	Identity as a singer (emotional connection with singing)
57	When I make plans, I think that I can make them work	Social inclusion
58	I don't like singing	Identity as a singer (emotional connection with singing)
59	Chance and luck are very important for what happens in my life	Social inclusion
60	I know how to be with other people	Social inclusion

The questionnaire was completed in the children's school class as part of a normal working day. The researcher led the session and the children's class teacher and teaching assistants provided support as necessary to ensure that the children worked on their own and were not copying anyone else. Children worked through the statements at their own speed and indicated their agreement with the statement using a seven-point Likert-type smiley face scale. Earlier studies had used versions of this type of visual analog scale to measure pain (e.g., Wong and Baker, 1988; Chapman and Kirby-Turner, 2002) and also anxiety and stress (e.g., Kindler et al., 2000; Preti, 2009). The initial research methods piloting revealed that this form of visual analog scale worked very well with young children, irrespective of academic ability, ethnicity, and language group. Individuals were supported in reading the text if this was required. For the youngest children, one page of the questionnaire was completed at a time (six questions), with the class teacher and teaching assistants supporting individual children as needed. Using Cronbach's alpha coefficient (α) as a measure of the internal reliability of children's answers, the overall questionnaire consistency for the instrument (60 items) was α = 0.87. The subset of *n* = 15 items for social inclusion (sense of self and of being socially integrated) responses was α = 0.68, which we recognize is not as high as the overall level, but still within the acceptable level, given the demographic and nature of the investigation.

A comparison of the two strands of collected data [i.e., individual normalized singing assessment data and the same children's mean questionnaire answers on their sense of social inclusion (sense of self and of being socially integrated)] enabled paired data to be matched for *n* = 6087 participants.

## Results

**Figure 1:**
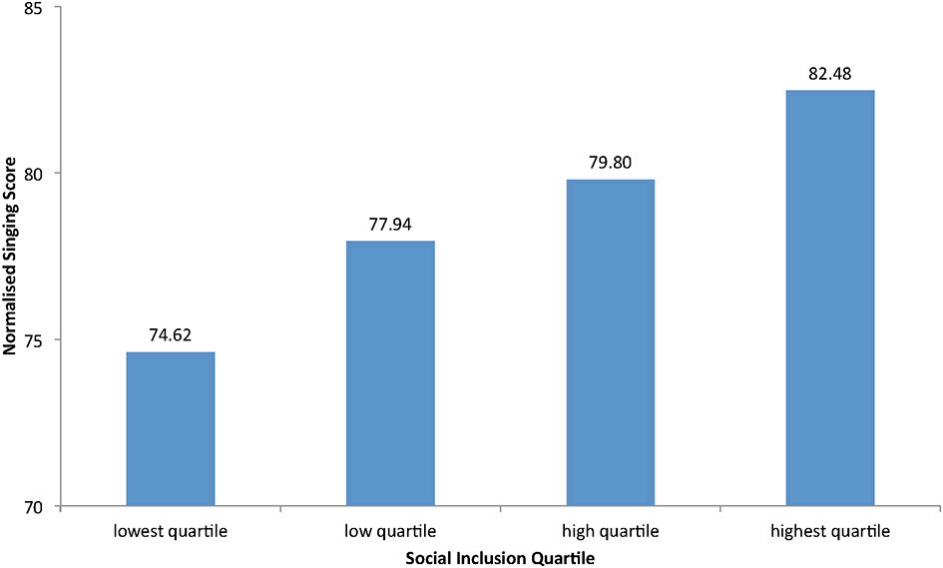
**A linear relationship is evidenced between participants' mean social inclusion (sense of self and of being socially integrated) responses in quartiles matched against the same individuals' normalized singing score (rated out of 100, where 100 equates to skilled in-tune singing and 50 is much less developmentally able, such as significant vocal pitch errors against the target melodies) for *n* = 6087 matched pairs**.

Initially, a Pearson product-moment correlation coefficient was computed to assess the relationship between the children's mean responses to the 15 questions concerning their social inclusion (sense of self and of being socially integrated) and the same children's individually assessed normalized singing scores. There was a small but significant correlation between the two variables, *r* = 0.089, *n* = 6.087, *p* < 0.0001 (two-tailed). Overall, there was a positive correlation between singing development rating and children's sense of being socially included. Increases in rated singing ability were correlated with increases in social inclusion. The relationship was also illustrated in a one-way ANOVA examining the connection between social inclusion scores, organized into quartiles, and measured singing ability, *F*_(3, 6083)_ = 5503.91, *p* < 0.0001 (see Figure 1). Children clustered within the highest quartile for their mean social inclusion scores had a mean normalized singing score of 82.48, in contrast to those in the lowest social inclusion quartile who had a mean normalized singing score of 74.62. *Post-hoc* Tukey analysis confirmed differences between each social inclusion quartile at *p* < 0.05 level of significance.

Subsequently, a series of regression analyses were undertaken. The results of the OLS regressions show that social inclusion (sense of self and of being socially integrated) score significantly predicted students' normalized singing score [β = 1.562, *t*_(6073)_ = 6.482, *p* < 0.001]. Thus, a 1-point increase in social inclusion score (on a 7-point scale) predicted a 1.562-point increase in singing score. These effects remained statistically significant and did not change substantially when controlling for gender, year group, and ethnicity. This was the case even though these demographic variables explained a significant proportion of variance in singing scores, and though each independent variable individually significantly predicted normalized singing scores [*R*^2^ = 0.147, *F*_(11, 6085)_ = 94.952, *p* < 0.001]^6^.

When dividing the social inclusion scale into quartiles, regression results showed that being in the lowest quartile for social inclusion predicted a 6.291-point decrease in singing score compared to being in the highest quartile [β = 6.291, *t*_(6073) = −2033_, *p* < 0.05]. Likewise, the regressions results showed that social inclusion scores explained a significant, albeit relatively low, proportion of variance in singing scores [*R*^2^ = 0.008, *F*_(1, 6085)_ = 48.69, *p* < 0.001].

The regression results also showed that intervention type significantly predicted students' normalized singing scores [β = 6.108, *t*_(6073)_ = 13.022, *p* < 0.001], even when demographic variables were controlled for. Thus, the results showed that being in a *Sing Up* school predicted an increase in singing scores of 6.108 points. Results also indicated that intervention type explained a significant proportion of variance in singing scores [*R*^2^ = 0.017, *F*_(1, 6085)_ = 104.363, *p* < 0.001].

In addition, as mentioned above, within the overall dataset, there were two main sub-groups. One sub-group consisted of children who were not participating in the *Sing Up* programme at the time of their assessment, but who completed both the attitudinal questionnaire and also undertook an individual singing assessment. This sub-group were termed *Non-Sing Up* in the data collection and subsequent analyses in order that they could be compared to the other sub-group of children who (at the time of their assessments) had had experience of the national singing programme (i.e., having been exposed to one or more of the *Sing Up* umbrella of activities). This second sub-group were labeled *Sing Up*. Consequently, additional analyses were undertaken of the *n* = 6087 matched pairs to examine if the overall correlation reported above was also evidenced for each of these two sub-groups. Two Pearson product-moment correlation coefficients were computed to assess the relationship between the children's responses to the questions concerning social inclusion (sense of self and of being socially integrated) and the same children's individual normalized singing score, sub-divided into *Non-Sing Up* (*n* = 1505) and *Sing Up* (*n* = 4582) participants. In each case, there was a small but identical significant positive correlation between the two variables, i.e., *Non-Sing Up r* = 0.093, *n* = 1505, *p* < 0.0001 and *Sing Up r* = 0.093, *n* = 4.582, *p* < 0.0001. Overall, a positive correlation was evidenced between children's sense of being socially included and their singing ability for each sub-group. There is evidence of a positive relationship between increased singing skill and a greater sense of self and of being socially included, whether or not children had participated in the *Sing Up* programme. This evidence of a relationship between measures for *both* groups confirms the interpretation that the development of singing expertise of itself, irrespective of *Sing Up* participation, appears to be related positively to self-concept (i.e., *Sing Up* participants were not being uniquely primed to be more positive about themselves through participation).

One further correlational analysis was undertaken. This was for children (irrespective of *Non-Sing Up/Sing Up* sub-group membership) for whom longitudinal matched pairs data were available. These particular children had completed the attitudinal questionnaire and had had their singing ability assessed at two different intervals during the final 3 years of the research team's main overall evaluation of the *Sing Up* programme (2008–2011). For these children (*n* = 666) it was possible to determine gain scores, being the difference between their original assessment data and their second assessment approximately 1 year later. A Pearson product-moment correlation coefficient was computed to assess the relationship between the children's gain scores related to any changes in their responses to questions concerning social inclusion (sense of self and of being socially integrated) compared to any changes over the same time period in the same children's individually assessed normalized singing scores. In line with the other findings reported above, there was a small but significant correlation between the two variables, *r* = 0.117, *n* = 666, *p* = 0.003, i.e., there was a tendency for increases in perceived social inclusion (sense of self and of being socially integrated) over time to be correlated longitudinally in these pupils with increases in ratings of singing ability.

## Discussion

The design and implementation of the National Singing Programme *Sing Up* in England was driven by a political concern at that time to ensure that all children of Primary school age experienced regular and successful singing experiences each week. In addition, underpinning this policy initiative, there was an official belief in the potential for music participation to generate wider benefits, as illustrated in such official statements as “… the power of music as an agent for personal, social and educational development” (Music Manifesto, 2006, p. 4), “The chance for our most vulnerable and marginalized children to change their lives through music” (op.cit. p24) and “Singing is a fast route to participative music making for every child and helps to build communities” (op.cit. p34). Notwithstanding the rhetorical style of such music advocacy, the data analyses arising from this external evaluation of *Sing Up* (as reported above) suggest that there is, at least, some empirical evidence of social as well as musical benefit from active participation in successful music making. Indeed, the regression results suggests that children with more advanced singing abilities were also more socially included. They also suggest that participation in the *Sing Up* programme was associated with a significant increase in singing scores. Together, these results suggest that contexts such as those offered by the *Sing Up* programme, i.e., where singing is fostered in a collective setting, can increase children's sense of social inclusion. In such contexts, many children acquire singing competence through participation in a group where learning is collaborative, satisfying, and significant, and where learning may be supported by group motivational processes, such as shared goals, the holding of positive outcome expectations, with the attribution of success associated with factors such as ability and effort [cf. drawing on Dweck's (1999) “incremental” perspective of development, rather than a fixed “entity” view of ability], as well as feeling efficacious about performing together. Additionally, social interactions between learners include the observation of peers and significant others who offer more competent and/or contrasting competency models. These are all strong motivational processes affecting self-efficacy and self-esteem (cf. Bandura's social cognitive theory where self-efficacy refers to “beliefs in one's capabilities to organize and execute the courses of action required to produce given attainments,” Bandura, 1997, p. 3). Whilst caution is needed in the interpretation of the correlation and regression data, and in the pitfalls of ascription of causal effects between measures, the inference is that successful singing is likely to be associated with a more positive sense of self because of perceived competence and singing self-efficacy which supports general self-esteem and the feeling of being social included. This social-psychological (including emotional) impact (where positive) also relates to the physical act of singing, its embodiment (cf. a key feature of music ensemble experience, McCaleb, 2014), that derives from early social experiences in the home and, subsequently, as part of collective experience in a communal (parent and toddler, then school) contexts.

Such an inference is supported by other research evidence, both arising as part of this national evaluation and elsewhere. For example, one strand of the national *Sing Up* programme, the *Chorister Outreach Programme (COP)*, was led by the Choir Schools Association. Their members were given the opportunity to bid for funds to initiate singing development activities (such as workshops and concerts) in their local Primary schools, often through the organization of visits by their choristers to act as singing role models, and usually with a senior member of the Cathedral music staff leading the school children's singing activities. A common outcome arising from the sequenced programme of weekly COP activities was a concert-type performance in the local Cathedral in which all the participant school children and choristers, together with parents and carers, came together. A research evaluation of the impact of this strand, using the same assessment tools and protocol outlined above, found that these *COP*-experienced Primary school children (*n* = 943) had the highest mean positive attitudes toward their sense of social inclusion (sense of self and of being socially integrated), the highest mean social inclusion scores compared to other sub-groupings within the dataset and amongst the highest mean singing assessment scores (Saunders et al., 2012). Furthermore, when the research team were asked to undertake a related evaluation for the Italian Ministry of Education of the social impact of their specially-funded choral programme in schools across the *Emilia-Romagna* region, similar findings emerged. Italian children who had experienced the previous year's choral programme (*n* = 98) had significantly higher mean social inclusion (sense of self and of being socially integrated) self-ratings compared to their peers (*n* = 92) who had not participated in the programme (Welch et al., 2010).

Drawing on the literature cited earlier and elsewhere, it is possible to speculate as to why children's successful engagement in singing might be associated in some way with an enhanced sense of self. For example, children have tended to learn to sing in large group settings within the English Primary school system. Singing is frequently experienced as a member of a whole class or within a group of classes, such as in a school assembly. As a result of the children's and their teachers' involvement in the national *Sing Up* programme, with its emphasis on group-based pedagogy, many children experienced growing mastery in their singing behavior and were developmentally in advance in their singing behaviors compared with children of the same age outside the programme (see Welch et al., 2012b, for an overview of the gendered impact). This is not to say that learning to sing as a member of a group automatically fosters individual development, but it seems that the design of the national programme, which sought to accommodate its group teaching bias by providing a rich range of on-line and paper resources, allowed for differentiated singing tasks that supported opportunities for successful teaching to be observed (Saunders et al., 2011).

In addition, other psychological research suggests that acting in synchrony with others can increase cooperation by strengthening social attachment among group members (Wiltermuth and Heath, 2009). This finding accords with evidence from adult studies concerning the important psychophysiological, socio-psychological, and well-being benefits that can accrue from choir membership (Bailey and Davidson, 2002; Faulkner and Davidson, 2006; Clift and Hancox, 2010; Clift et al., 2010), including evidence of an increase in positive affect (mood enhancement) and participants' immune response (Kreutz et al., 2004; see also Chanda and Levitin, 2013, for a review). Similarly, research evidence from adolescent engagement in other arts areas, such as dance, and also sport suggests that peer relationships can be a powerful factor in nurturing (or hindering) successful participation and ongoing engagement (Patrick et al., 1999; Aujla et al., 2013), a finding related to social benefit that is also evidenced in adolescent music activity, both in school and elsewhere (Saunders and Welch, 2012). Similarly, narrative based research enquiry into adolescent boys' motivation to continue singing activities is reported to relate to their self-perceptions of musical autonomy and vocal skills that are nurtured within a network of peer social support (Freer, 2009). The finding on the power of synchrony can be seen also to provide the foundation for the experience of “empathic creativity” in music making with others (Cross et al., 2012), whereby social interaction through music is possible, not least because music can be seen as a social behavior. Similarly, complementary neuroscientific dual-fMRI-based evidence indicates that singing with another person, such as in a duet, involves a distributed network of brain areas that are responsible for coordinating interactive entrainment (Parsons et al., 2009). Consequently, where children experience success in the context of their collective singing, with associated feelings related to emotional and social well-being as part of an underlying distributed neural network, it is not surprising that they might report a stronger sense of group membership, of belonging and of being social included.

In conclusion, the three separate, yet complementary strands of correlational data within the emergent analyses from the two strands of assessment (singing behavior and social inclusion i.e., sense of self and of being socially integrated) undertaken with this large participant group of children aged 7+ to 10+ appear to support previous evidence that singing can be beneficial in building a sense of community. Children with more developed singing ability (irrespective of whether or not they had any experience of *Sing Up*) tended to have a more positive sense of self and of being socially integrated. Where children had experience of the *Sing Up* programme, they were statistically more likely to be advanced in their singing development compared to those children outside the programme. By inference, therefore, the programme was also indirectly providing social benefit (i.e., as defined by this research instrument). Whilst singing is not being proposed here as a panacea for the many families “at risk” in our societies, the public's interest in singing (as judged by current television media programmes, music sales across a wide range of platforms, and membership of choirs) opens up the possibility for this form of personal yet social music making to have a positive contribution to children's self-concept and wellbeing.

### Conflict of interest statement

The authors declare that the research was conducted in the absence of any commercial or financial relationships that could be construed as a potential conflict of interest.

## Footnotes

^1^See http://www.singup.org/about-us/history/music-manifesto/ originally retrieved 21 August 2009.

^2^See http://www.singup.org/ originally retrieved 2 September 2009. The incoming coalition Government subsequently extended the programme for one further year (2011–2012) at an additional cost of £4 m.

^3^Children's experience of Sing Up could be in one or more of several strands within the programme, such as (a) their teachers (class teachers or community musicians) participating in the national Sing Up “workforce development” programme to improve the teaching of singing in schools and community settings; and/or (b) using a new specially designed on-line resource to support singing teaching and learning—the Song Bank; and/or having sustained specialist singing teaching input in their school—such as provided by the Choir School Association's Chorister Outreach Programme or Ex Cathedra's Singing Playgrounds initiative.

^4^This bias in the size of the two groupings was a product of the way that the national programme was rolled out across the country. With each successive year of data collection, more and more schools joined the programme. Consequently, much of the Non Sing Up data was collected in the opening 2 years of the research process. It was estimated that over 95% of state Primary schools in England were participating in the Sing Up programme by March 2011 (Sing Up, 2011).

^5^The six themes in the children's questionnaire were: (1) Identity as a singer (focusing on emotional connection with singing); (2) Identity as a singer (related to self-efficacy); (3) Singing at home; (4) Singing at school; (5) Singing in informal settings; and (from the second year of data collection onwards); (6) Self-concept and sense of social inclusion.

^6^Separate analyses of children's normalized singing scores revealed that girls tend to be more advanced in their singing than boys, Black and White children tend to be more advanced in their singing than Asian children, older children tend to be more advanced in their singing than younger children, and that, overall, children with experience of *Sing Up* were more advanced in their singing than children outside the programme (Welch et al., 2012b; Welch et al., under review).

## References

[B1] Achenbach T. M. (1991). Integrative Guide for the 1991 CBCL/4-18, YSR and TRF Profiles. Burlington: Department of Psychiatry, University of Vermont

[B2] Agilis S. A. (2012). Lot 1: EU-SILC (European Union Statistics on Income and Living Conditions): methodological studies and publications, in Working paper on the Description of the ‘Income and Living Conditions Dataset’ (Athens: Agilis S.A.).

[B3] Alexiadou N. (2002). Social inclusion and social exclusion in England: tensions in education policy. J. Educ. Policy 17, 71–86 10.1080/02680930110100063

[B4] Allman D. (2013). The sociology of social inclusion. Sage Open 3, 1–16 10.1177/2158244012471957

[B5] Atkinson T. Cantillon B. Marlier E. Nolan B. (2002). Social Indicators: the EU and Social Inclusion. Oxford: Oxford University Press 10.1093/0199253498.001.0001

[B6] Aujla I. J. Nordin-Bates S. M. Redding E. (2013). A qualitative investigation of commitment to dance: findings from the UK centres for advanced training. Res. Dance Educ. 15, 138–160 10.1080/14647893.2013.825764

[B7] Australian Social Inclusion Board. (2010). Social Inclusion in Australia: How Australia is Faring. Canberra: Department of the Prime Minister and Cabinet

[B8] Bailey B. Davidson J. W. (2002). Adaptive characteristics of group singing: perceptions from members of a choir for homeless men. Music. Sci. 2, 221–256 10.1177/102986490200600206

[B9] Bandura A. (1997). Self-Efficacy: The Exercise of Control. New York, NY: W. H. Freeman

[B10] Barrett M. S. Baker J. S. (2012). Developing learning identities in and through music: a case study of the outcomes of a music programme in an Australian juvenile detention centre. Int. J. Music Educ. 30, 244–259 10.1177/0255761411433721

[B11] Bartz J. A. Hollander E. (2006). The neuroscience of affiliation: forging links between basic and clinical research on neuropeptides and social behavior. Horm. Behav. 50, 518–528 10.1016/j.yhbeh.2006.06.018 16884725

[B12] Baumeister R. F. DeWall C. N. Ciarocco N. J. Twenge J. M. (2005). Social exclusion impairs self-regulation. J. Pers. Soc. Psychol. 88, 589–604 10.1037/0022-3514.88.4.589 15796662

[B13] BERA. (2004). Revised Ethical Guidelines for Educational Research. Southwell: BERA

[B14] Boer D. (2009). Music Makes the People Come Together: Social Functions of Music Listening for Young People Across Cultures. Published Ph.D. Thesis (Victoria University of Wellington). Available online at: http://researcharchive.vuw.ac.nz/bitstream/handle/10063/1155/thesis.pdf?sequence=1 (Accessed November 1, 2013).

[B15] Brown E. D. Benedett B. Armistead M. E. (2010). Arts enrichment and school readiness for children at risk. Early Child. Res. Q. 25, 112–124 10.1016/j.ecresq.2009.07.008

[B16] Catterall J. S. (2009). Doing Well and Doing Good by Doing Art: A 12-Year Longitudinal Study of Arts Education—Effects on the Achievements and Values of Young Adults. Los Angeles, CA: I-Group Books

[B17] Catterall J. S. Chapleau R. Iwanaga J. (1999). Involvement in the arts and human development: general involvement and intensive involvement in music and theatre arts, in Champions of Change: The Impact of the Arts on Learning, ed Fiske E. B. (Washington DC: President's Committee on the Arts and Humanities/Arts Education Partnership), 1–18 Available online at: http://artsedge.kennedy-center.org/champions/pdfs/ChampsReport.pdf (Accessed February 17, 2014).

[B18] Chanda M. L. Levitin D. J. (2013). The neurochemistry of music. Trends Cogn. Sci. 17, 179–193 10.1016/j.tics.2013.02.007 23541122

[B18a] Chapman H. R. Kirby-Turner N. (2002). Visual/verbal analogue scales: examples of brief assessment methods to aid management of child and adult patients in clinical practice. Br. Dent. J. 193, 447–450 10.1038/sj.bdj.4801593 12516670

[B19] Chong H. J. (2010). Do we all enjoy singing? A content analysis of non-vocalists' attitudes towards singing. Arts Psychother. 37, 120–124 10.1016/j.aip.2010.01.001

[B20] Chorus America. (2003). America's Performing Art: A Study of Choruses, Choral Singers, and their Impact. Washington, DC: Chorus America

[B21] Cirelli L. Einarson K. M. Lade S. Trainor L. J. (2013). Interpersonal motor synchrony to a musical beat as a cue for social cohesion during infancy, in Conference Abstract: 14th Rhythm Production and Perception Workshop Birmingham 11th - 13th September 2013. Front. Hum. Neurosci. (Birmingham). 10.3389/conf.fnhum.2013.214.00018

[B22] Clift S. Hancox G. (2010). The significance of choral singing for sustaining psychological wellbeing: findings from a survey of choristers in England, Australia and Germany. Music Perform. Res. 3, 79–96

[B23] Clift S. Hancox G. Morrison I. Hess B. Kreutz G. Stewart D. (2010). Choral singing and psychological wellbeing: quantitative and qualitative findings from english choirs in a cross-national survey. J. Appl. Arts Health 1, 19–34 10.1386/jaah.1.1.19/1

[B24] Clift S. Hancox G. Morrison I. Hess B. Stewart D. Kreutz G. (2008). Choral Singing, Wellbeing and Health: Findings from a Cross National Survey. Canterbury: Canterbury Christ Church University. Available online at: https://www.canterbury.ac.uk/Research/Centres/SDHR/Documents/ChoralSingingSummary.pdf (Accessed November 1, 2013).

[B25] Costa-Giomi E. (2004). Effects of three years of piano instruction on children's academic achievement, school performance and self-esteem. Psychol. Music 32, 139–152 10.1177/0305735604041491

[B26] Creech A. Gonzales-Moreno P. Lorenzino L. with Bates L. Swan A. de Jesus Carillo Mendez R. (2013a). El Sistema and Sistema-Inspired Programmes: A Literature Review of Research, Evaluation and Critical Debates. San Diego, CA: Sistema Global

[B27] Creech A. Hallam S. McQueen H. Varvarigou M. (2013b). The power of music in the lives of older adults. Res. Stud. Music Educ. 35, 87 10.1177/1321103X13478862

[B28] Croom A. M. (2012). Music, neuroscience and the psychology of well-being: a précis. Front. Psychol. 2:393 10.3389/fpsyg.2011.00393 22232614 PMC3249389

[B29] Cross I. Laurence F. Rabinowitch T. L. (2012). Empathy and creativity in group musical practices: towards a concept of empathic creativity, in The Oxford Handbook of Music Education, Vol. II, eds McPherson G. Welch G. F. (New York, NY: Oxford University Press), 337–353

[B30] Department for Work Pensions (DWP). (2012). National Social Report. London: Department for Work and Pensions

[B31] DFE. (2011). The Importance of Music. A National Plan for Music Education. London: Department for Education/Department for Media, Culture and Sport

[B32] Dingle G. A. Brander C. Ballantyne J. Baker F. A. (2013). ‘To be heard’: the social and mental health benefits of choir singing for disadvantaged adults. Psychol. Music 41, 405–421 10.1177/0305735611430081

[B33] Dissanayake E. (2008). If music is the food of love, what about survival and reproductive success? Musicae Scientiae, 169–195 10.1177/1029864908012001081

[B34] Dweck C. S. (1999). Self-Theories: Their Role in Motivation, Personality, and Development. Philadelphia, PA: The Psychology Press 2130257

[B36] EC. (2009). Social Protection and Social Inclusion 2008: EU Indicators. Luxembourg: Office for Official Publications of the European Communities

[B37] EC. (2013). Social Europe: Current Challenges and the Way Forward-Annual Report of the Social Protection Committee (2012). Luxembourg: Publications Office of the European Union

[B38] Eccles J. Wigfield A. Harold R. D. Blumenfeld P. (1993). Age and gender differences in children's self- and task perceptions during elementary school. Child Dev. 64, 830–847 10.2307/1131221 8339698

[B35] EU-SILC. (2006). European Union Statistics on Income and Living Conditions [EU-SILC]. Luxembourg: Eurostat 10.1093/ije/dyv069 25948659

[B39] Freeman W. J. III (2000). A neurobiological role of music in social bonding, in The Origins of Music eds Wallin N. Merkur B. Brown S. (Cambridge MA: MIT Press), 411–424 Available online at: http://escholarship.org/uc/item/9025x8rt

[B40] Faulkner R. Davidson J. W. (2006). Men in chorus: collaboration and competition in homo-social vocal behaviour. Psychol. Music 34, 219–237 10.1177/0305735606061853

[B41] Ferguson K. M. (2006). Social capital and children's wellbeing: a critical synthesis of the international social capital literature. Int. J. Soc. Welf. 15, 2–18 10.1111/j.1468-2397.2006.00575.x

[B42] Fitts W. H. (1964). The Fitts Tennessee Self Concept Scale Questionnaire. Los Angeles, CA: Western Psychological Services [Updated Fitts W. H. Warren W. L. (1991). Tennessee Self-Concept Scale Second Edition (TSCS:2), Los Angeles, CA: Western Psychological Services]

[B43] Frazer H. Marlier E. (2011). Assessment of Progress Towards the Europe 2020 Social Inclusion Objectives. Brussels: European Commission DG Employment, Social Affairs and Inclusion Available online at: http://www.peer-review-social-inclusion.eu (Accessed November 1, 2013).

[B44] Frederickson N. L. Furnham A. F. (2001). The long-term stability of sociometric status classification: a longitudinal study of included pupils who have moderate learning difficulties and their mainstream peers. J. Child Psychol. Psychiatry 42, 581–592 10.1111/1469-7610.00754 11464963

[B45] Fredrikson M. Welch G. Porras J. Paananen P. Read J. C. Stadler Elmer S. (2009). Music as an enabler for social inclusion and provision - The UMSIC approach, in Assistive Technology from Adapted Equipment to Inclusive Environments, eds Emiliani P. L. Burzagli L. Como A. Gabbani F. Salminen A. L. (Amsterdam: IOS Press), 622–627

[B46] Freer P. (2009). Boys' descriptions of their experiences in choral music. Res. Stud. Music Educ. 31, 142–160 10.1177/1321103X09344382

[B47] Haeberlin U. Moser U. Bless G. Klaghofer R. (1989). Integration in die Schulklasse: Fragebogen zur Erfassung von Dimensionen der Integration von Schülern, FDI 4-6 (Beiträge zur Heil- und Sonderpädagogik). Bern: Paul Haupt

[B48] Hampshire K. R. Matthijsse M. (2010). Can arts projects improve young people's wellbeing? A social capital approach. Soc. Sci. Med. 71, 708–716 10.1016/j.socscimed.2010.05.015 20579795

[B49] Hanna G. Patterson M. Rollins J. Sherman A. (2011). The Arts And Human Development: Framing a National Research Agenda For The Arts, Lifelong Learning, And Individual Well-Being. Washington, DC: National Endowment for the Arts

[B50] Harvey F. McNeilly N. (2012). Global Audit of National Youth Orchestra, El Sistema and Sistema-Inspired Initiatives and Youth Orchestra Networks. London: British Council

[B51] Henley J. Caulfield L. S. Wilson D. (2012). Good Vibrations: positive change through social music-making. Music Educ. Res. 14, 499–520 10.1080/14613808.2012.714765

[B52] JMI. (2010). Making a Difference Through Music: Annual Report 2010. Brussels: Jeunesses Musicales International

[B52a] Kindler C. Harms C. Amsler F. Ihde-Scholl T. Scheidegger D. (2000). The visual analogue scale allows effective measurement of preoperative anxiety and detection of patients' anaesthetic concerns. Anesth. Analg. 90, 706–712 10.1097/00000539-200003000-00036 10702461

[B53] Kreutz G. Bongard S. Rohrmann S. Hodapp V. Grebe D. (2004). Effects of choir singing or listening on secretory immunoglobulin a, cortisol and emotional state. J. Behav. Med. 27, 623–635 10.1007/s10865-004-0006-9 15669447

[B54] Langston T. W. Barrett M. S. (2008). Capitalizing on community music: a case study of the manifestation of social capital in a community choir. Res. Stud. Music Educ. 30, 118–138 10.1177/1321103X08097503

[B55] MacDonald R. A. R. (2013). Music, health, and well-being: a review. Int. J. Qual. Stud. Health Well Being 8:20635 10.3402/qhw.v8i0.20635 23930991 PMC3740599

[B56] MacDonald R. Kreutz G. Mitchell L. (eds.). (2012). Music, Health, and Wellbeing. London: Oxford University Press 10.1093/acprof:oso/9780199586974.001.0001

[B57] Mang E. (2006). The effects of age, gender and language on children's singing competency. Br. J. Music Educ. 23, 161–174 10.1017/S0265051706006905

[B58] Mannion G. (2003). Children's participation in school grounds developments: creating a place for education that promotes children's social inclusion. Int. J. Inclusive Educ. 7, 175–192 10.1080/13603110304784

[B59] McCaleb J. M. (2014). Embodied Knowledge in Ensemble Performance. Farnham: Ashgate

[B60] McPherson G. E. McCormick J. (2006). Self-efficacy and music performance. Psychol. Music 34, 332–336 10.1177/0305735606064841

[B61] Micklewright J. (2002). Social Exclusion and Children: a European View for a US Debate. Florence: United Nation's Children's Fund

[B62] Music Manifesto. (2006). Making Every Child's Music Matter. Music Manifesto Report no. 2. A Consultation for Action. London: Department for Education and Skills

[B63] Myllykoski M. Paananen P. (2009). Towards New Social Dimensions for Children's Music Making - JAMMO as a Collaborative and Communal M-Learning Environment. Available online at: http://urn.fi/URN:NBN:fi:jyu-2009411301 (Accessed November 1, 2013).

[B64] Nelson E. E. Leibenluft E. McLure E. B. Pine D. S. (2005). The social re-orientation of adolescence: a neuroscience perspective on the process and its relation to psychopathology. Psychol. Med. 35, 163–174 10.1017/S0033291704003915 15841674

[B65] Noice H. Noice T. (2009). An arts intervention for older adults living in subsidized retirement homes. Aging Neuropsychol. Cogn. 16, 56–79 10.1080/13825580802233400 18686051 PMC2769921

[B66] Nowicki S. Strickland B. R. (1973). A locus of control scale for children. J. Consult. Clin. Psychol. 40, 148–154 10.1037/h0033978 4694214

[B67] Nowicki S. Walker C. (1973). The role of generalised and specific expectancies in determining academic achievement. J. Soc. Psychol. 94, 275–280 10.1080/00224545.1974.9923214

[B68] O'Dougherty Wright M. Masten A. S. Narayan A. J. (2013). Resilience processes in development: four waves of research on positive adaptation in the context of adversity, in Handbook of Resilience in Children, eds Goldstein S. Brooks R. B. (New York, NY: Springer), 15–37 10.1007/978-1-4614-3661-4_2

[B69] Papousek H. (1996). Musicality in infancy research: biological and cultural origins of early musicality, in Musical Beginnings, eds Deliege I. Sloboda J. (Oxford: Oxford University Press), 37–55 10.1093/acprof:oso/9780198523321.003.0002

[B70] Parsons L. M. Himonides E. Craig N. Vakil M. Turner R. Wilkinson I. (2009). Simultaneous dual-fMRI, sparse temporal scanning of human duetters at 1.5 and 3 Tesla. Proc. Int. Soc. Magn. Reson. Med. 17, 3711

[B71] Patrick H. Ryan A. M. Alfeld-Liro C. Fredricks J. A. Hruda L. Z. Eccles J. S. (1999). Adolescents commitment to developing talent: the role of peers in continuing motivation for sports and the arts. J. Youth Adolesc. 28, 741–763 10.1023/A:1021643718575

[B72] Plumridge J. M. (1972). The Range and Pitch Levels of Children's Voices, in Relation to Published Material for Children's Voices. Unpublished Dissertation, Diploma of Advanced Study of Education, University of Reading, Reading.

[B73] Portowitz A. Lichtenstein O. Egorow L. Brand E. (2009). Underlying mechanisms that connect music education and cognitive modifiability. Res. Stud. Music Educ. 31, 107–128 10.1177/1321103X09344378

[B73a] Preti C. (2009). Music in Hospitals: Anatomy of a Process. Unpublished Ph.D. Thesis. Institute of Education, University of London.

[B74] Preti C. (2013). Live music as a bridge between paediatric hospitals and outside communities: a proposed research framework and a review of the literature. UNESCO Obser. Multi-Discip. J. Arts 3, 1–18

[B75] Preti C. Welch G. F. (2011). Music in a hospital: the impact of a live music program on pediatric patients and their caregivers. Music Med. 3, 213–223 10.1177/1943862111399449

[B76] Rabinowitch T.-C. Cross I. Burnard P. (2013). Long-term musical group interaction has a positive influence on empathy in children. Psychol. Music 41, 484–498 10.1177/0305735612440609

[B77] Rickard N. S. Appleman P. James R. Murphy F. Gill A. Bambrick C. (2013). Orchestrating life skills: the effect of increased school-based music classes on children's social competence and self-esteem. Int. J. Music Educ. 31, 292–309 10.1177/0255761411434824

[B78] Rinta T. Purves R. Welch G. F. (2011b). Usability of a Jamming Mobile with 3-6 year-old children for enhancing feelings of social inclusion and facilitating musical learning, in Proceedings of the 5th Conference of the European Network of Music Educators and Researchers of Young Children (Helsinki), 275–286

[B79] Rinta T. Purves R. Welch G. Stadler Elmer S. Bissig R. (2011a). Connections between children's feelings of social inclusion and their musical backgrounds. J. Soc. Inclusion 2, 2

[B80] Ritchie L. Williamon A. (2011). Primary school children's self-efficacy for music learning. J. Res. Music Educ. 59, 146–161 10.1177/0022429411405214

[B81] Rosenberg M. (1989). Society and the Adolescent Self-Image, Revised Edition. Middletown, CT: Wesleyan University Press

[B82] Rutkowski J. (1997). The nature of children's singing voices: characteristics and assessment, in The Phenomenon of Singing, ed Roberts. B. A. (St. John's, NF: Memorial University Press), 201–209

[B83] Ryan R. M. Deci E. L. (2000). Intrinsic and extrinsic motivations: classic definitions and new directions. Contemp. Educ. Psychol. 25, 54–67 10.1006/ceps.1999.1020 10620381

[B84] Saunders J. Papageorgi I. Himonides E. Rinta T. Welch G. F. (2011). Researching the Impact of the National Singing Programme ‘Sing Up’ in England: Diverse Approaches to Successful Singing in Primary Settings. London: International Music Education Research Centre, Institute of Education

[B85] Saunders J. Papageorgi I. Himonides E. Vraka M. Rinta T. Welch G. F. (2012). The Chorister Outreach Programme of the Choir Schools Association. London: International Music Education Research Centre, Institute of Education

[B86] Saunders J. Welch G. F. (2012). Communities of Music Education. London: Youth Music/International Music Education Research Centre, Institute of Education

[B87] Schmidt C. P. Zdzinski S. F. Ballard D. L. (2006). Motivation orientations, academic achievement, and career goals of undergraduate music education majors. J. Res. Music Educ. 54, 138–153 10.1177/002242940605400205

[B88] Sing Up (2011). Sing Up Synthesis Report. London: Sing Up Available online at: http://www.musikk.no/sfiles/9/83/50/66/5/file/sing_up_evaluation_synthesis_report.pdf (Accessed November 1, 2013).

[B89] Siraj-Blatchford I. Mayo A. Melhuish E. Taggart B. Sammons P. Sylva K. (2011). Performing Against the Odds: Developmental Trajectories of Children in the EPPSE 3-16 Study. London: Department for Education [Research Report DFE-RR128.]

[B90] Smeijesters H. Kil J. Kurstjens H. Welten J. Willemars G. (2011). Arts therapies for young offenders in secure care—a practice-based research. Arts Psychother. 38, 41–51 10.1016/j.aip.2010.10.005

[B91] Teater B. Baldwin M. (2014). Singing for successful ageing: the perceived benefits of participating in the golden oldies community-arts programme. Br. J. Soc. Work 44, 81–99 10.1093/bjsw/bcs095

[B92] Thornberry T. P. Lizotte A. J. Krohn M. D. Farnworth M. Jang S. J. (1994). Delinquent peers, beliefs, and delinquent behavior: a longitudinal test of interactional theory. Criminology 32, 47–83 10.1111/j.1745-9125.1994.tb01146.x

[B93] Trevarthen C. (2008). The musical art of infant conversation: narrating in the time of sympathetic experience, without rational interpretation, before words. Musicae Scientiae, 15–46 10.1177/1029864908012001021

[B94] Twenge J. Baumeister R. DeWall C. Ciarocco N. Bartels J. (2007). Social exclusion decreases prosocial behavior. J. Pers. Soc. Psychol. 92, 56–66 10.1037/0022-3514.92.1.56 17201542

[B95] UN General Assembly. (2013). A Life of Dignity For All: Accelerating Progress Towards the Millennium Development Goals and Advancing the United Nations Development Agenda Beyond 2015. A Report of the Secretary-General. New York, NY: United Nations Available online at: http://www.un.org/millenniumgoals/pdf/ALifeofDignityforAll.pdf (Accessed February 1, 2014).

[B96] Vaughan T. Harris J. Caldwell B. J. (2011). Bridging the Gap in School Achievement Through the Arts. Abbotsford, BC: The Song Room

[B97] Vispoel W. P. (1994). Integrating self-perceptions of musical skill into contemporary models of self-concept. Quarterly 5, 42–57

[B98] Welch G. F. (1986). A developmental view of children's singing. Br. J. Music Educ. 3, 295–303 10.1017/S0265051700000802

[B99] Welch G. F. (1998). Early childhood musical development. Res. Stud. Music Educ. 11, 27–41 10.1177/1321103X9801100104

[B100] Welch G. F. (2006). Singing and vocal development, in The Child as Musician: A Handbook of Musical Development, ed McPherson G. (New York, NY: Oxford University Press), 311–329

[B101] Welch G. F. (2009). Evidence of the development of vocal pitch matching ability in children. Jpn. J. Music Educ. Res. 39, 38–47

[B102] Welch G. F. Himonides E. Saunders J. Papageorgi I. Rinta T. Preti C. (2012a). Researching the first year of the national singing programme in England: an initial impact evaluation. Psychomusicol. Music Mind Brain. 21, 83–97 10.1037/h0094006

[B103] Welch G. F. Preti C. Himonides E. (2010). Didattica della vocalita e della coratita, in Musica–Dalla Indicazioni Alla Practica Didattrica, ed Toni B. (Naples: Tecnondid Editrice), 22–25

[B104] Welch G. F. Saunders J. Papageorgi I. Himonides E. (2012b). Sex, gender and singing development: making a positive difference to boys' singing through a national programme in England, in Perspectives on Males and Singing, eds Harrison S. Welch G. F. Adler A. (London: Springer), 37–54

[B105] Welch G. F. Sergeant D. C. White P. (1997). Age, sex and vocal task as factors in singing ‘in-tune’ during the first years of schooling. Bull. Council Res. Music Educ. 133, 153–160

[B106] Wiggins J. H. (2007). Compositional process in music, in International Handbook of Research in Arts Education, ed Bresler L. (Dordrecht: Springer), 453–467 10.1007/978-1-4020-3052-9_29

[B107] Wiltermuth S. S. Heath C. (2009). Synchrony and cooperation. Psychol. Sci. 20, 1–5 10.1111/j.1467-9280.2008.02253.x 19152536

[B107a] Wong D. Baker C. (1988). Pain in children: comparison of assessment scales. Pediatr Nurs. 11, 9–17 3344163

[B108] Young S. (2003). Music With the Under-Fours. London: RoutledgeFalmer

